# What Can Surgery Do?

**Published:** 1899-04-22

**Authors:** 


### WHAT CAN SURGERY DO?

From a practical point of view by far the most interesting papers among those we are considering are those dealing with the questions: What can surgery do to relieve sufferers from cancer in various parts of the body? and, What treatment is still available in cases in which operation is impossible? The first of these is dealt with by Mr. Watson Cheyne and the second by Dr. William B. Coley, of New York. Mr. Cheyne holds strongly to the local origin of cancer and to the possibility of curing it by operation, if this be done sufficiently early and if it be carried out in such a manner as really to remove the diseased tissues. He says that the view taken of the question of the possible eradication of cancer by operation depends on the view taken of the nature of the disease, namely, whether it is essentially a local, or a constitutional trouble, in the first instance. His own view is that it is at first essentially a local overgrowth of one or a few epithelial cells which acquire parasitic properties and great powers of reproduction, and are then able to penetrate into the tissues in all directions, especially along the lymph channels, and destroy them, and before very long spread to internal organs and produce similar growths there. A varying interval of time elapses before these cells get beyond reach, and if a free removal of the infected area be carried out before such extension has taken place, the patient may be absolutely cured of the disease. Cancer cells are, he says, evidently derived from the epithelium of the part in which they originate. If they appear in the skin they resemble squamous epithelium; if in mucous membrane which is covered with cylindrical epithelial cells, they are cylindrical cells possessing the same characteristics and tendencies as the cylindrical cells in the part; if in the breast they possess the character of the epithelium of the breast; and so on. There is, in fact, no evidence that a cancer can *originate* elsewhere than from epithelium. A further and decisive argument that cancer is at first a local disease is the fact that after complete removal patients may remain free from recurrence for a long time, and indeed up to their death from other causes. Cases are on record where even 40 years have elapsed without any further trace of cancer, and he mentions that he has no less than 11 patients now alive at periods of from six to nine and a-half years after operation, without a trace of any recurrence, forming about 50 per cent. of all the cases operated on during that period. He does not, however, maintain that there may not be a greater tendency to cancer in some people than in others, and that even if one cancer be got rid of another may not be more likely to appear than in a patient who had not been so affected. This is a point that constantly complicates the question whether a patient is really cured by operation, since it is not every second cancer that can fairly be called a recurrence. As is well known, Volkmann has suggested three years as a fair average limit, after which, if no recurrence has taken place, a patient may be regarded as cured; and Mr. Watson Cheyne says that in patients quite free from disease at the end of three years, after an extensive operation performed on the principles laid down by Heidenhain and Stiles, he feels very hopeful, and that, as a matter of fact, taking patients perfectly well, without any trace of recurrence at the end of even one year, after the new operation for cancer of the breast, his statistics give the chances in favour of non-recurrence as about seventeen to one.

As for the treatment of inoperable cases, hopeless as it may often seem, it is well to be fully alive to what is possible. Dr. Coley, writing on this subject, refers to the following methods: Ovariectomy, combined with the administration of thyroid extract; the administration of chelidonium majus (celandine); electricity; kataphoresis; parenchymatous injection of alcohol; carbide of calcium; the administration of lymph gland extract; and the treatment of inoperable sarcoma by the mixed toxins of erysipelas and bacillus prodigiosus, the latter being the process with which his name is especially associated. We are surprised to find no mention of the systematic administration of opium. We cannot, however, but feel that all these methods are but broken reeds.

